# Retraction Note: Exemestane blocks mesothelioma growth through downregulation of cAMP, pCREB and CD44 implicating new treatment option in patients affected by this disease

**DOI:** 10.1186/s12943-022-01692-9

**Published:** 2022-12-09

**Authors:** Barbara Nuvoli, Sabrina Germoni, Carlotta Morosetti, Raffaela Santoro, Giancarlo Cortese, Serena Masi, Iole Cordone, Rossella Galati

**Affiliations:** 1 Molecular Medicine Area, Regina Elena National Cancer Institute, 00144 Rome, Italy; 2 S.A.F.U. Department, Regina Elena National Cancer Institute, Rome, Italy; 3 Clinical Pathology, Regina Elena National Cancer Institute, Rome, Italy

**Retraction Note: Mol Cancer 13, 69 (2014)**

**https://doi.org/10.1186/1476-4598-13-69**

The Editor-in-Chief has retracted this article. After publication, concerns were raised regarding the cell and western blot images presented in the figures. Specifically:

- In Fig. 1D, the NCI and MSTO EXE group images appear highly similar; the authors have confirmed that the original image for MSTO EXE has been replaced in the figure.
- The NCI and Met-5A images in Fig. 1D are also highly similar to HFF and Met5A in Fig. 2 in [1].
- Fig. 1G EXE (48) MSTO and CNTR (0) NCI images appear highly similar to those in Fig. 2D (48) MSTO and NCI, respectively (rotated).
- In Fig. 2B, the western blot images are composites of multiple gels without clear indication of image cropping or editing.

Additionally, the ethics approval information provided by the authors upon the Journal's request does not match the details stated in the Methods section.

The Editor-in-Chief therefore no longer has confidence in the presented data.

Rosella Galati does not agree to this retraction. The Publisher has not been able to obtain a current email address for Barbara Nuvoli, Sabrina Germoni, Carlotta Morosetti, Raffaela Santoro, Giancarlo Cortese, Serena Masi and Iole Cordone.
